# Nationwide hospital‐based survey of idiopathic normal pressure hydrocephalus in Japan: Epidemiological and clinical characteristics

**DOI:** 10.1002/brb3.635

**Published:** 2017-01-27

**Authors:** Nagato Kuriyama, Masakazu Miyajima, Madoka Nakajima, Michiko Kurosawa, Wakaba Fukushima, Yoshiyuki Watanabe, Etsuko Ozaki, Yoshio Hirota, Akiko Tamakoshi, Etsuro Mori, Takeo Kato, Takahiko Tokuda, Akinori Urae, Hajime Arai

**Affiliations:** ^1^ Department of Epidemiology for Community Health and MedicineKyoto Prefectural University of MedicineKyotoJapan; ^2^ Department of NeurologyKyoto Prefectural University of MedicineKyotoJapan; ^3^ Department of NeurosurgeryJuntendo University Graduate School of MedicineTokyoJapan; ^4^ Department of Epidemiology and Environmental HealthJuntendo University Graduate School of MedicineTokyoJapan; ^5^ Department of Public HealthOsaka City University Faculty of MedicineOsakaJapan; ^6^ College of Healthcare ManagementFukuokaJapan; ^7^ Department of Public HealthHokkaido University Graduate School of MedicineSapporoJapan; ^8^ Department of Behavioral Neurology and Cognitive NeuroscienceTohoku University Graduate School of MedicineSendaiJapan; ^9^ Department of Neurology, Hematology, Metabolism, Endocrinology and DiabetologyYamagata University Faculty of MedicineYamagataJapan; ^10^ Mediscience Planning Inc.TokyoJapan

**Keywords:** idiopathic normal pressure hydrocephalus, nationwide survey, clinical characteristics, comorbidity

## Abstract

**Objectives:**

There have been no nationwide epidemiological studies of idiopathic normal pressure hydrocephalus (iNPH) in Japan. Therefore, a nationwide epidemiologic survey of iNPH was performed to determine the number of cases and clinical characteristics by sex and diagnostic level.

**Methods:**

The first survey examined the numbers of cases that met the diagnostic criteria of iNPH and those who underwent shunt operations in 2012. The second survey gathered patients' details to clarify their clinical background characteristics.

**Results:**

The estimated number of cases meeting the diagnostic criteria in 2012 was 12,900, with 6,700 undergoing shunt operations. The estimated crude prevalence was 10.2/100,000 persons. The age of onset was in the 70s in more than 50% of both men and women. Significantly higher (*p *< .05) frequencies of gait impairment in men and cognitive decline in women were observed as initial symptoms. At the time of definitive diagnosis, gait impairment was observed most frequently in patients with definite iNPH (77.7%). Hypertension was the most frequent comorbidity (40.0%), followed by diabetes mellitus (17.8%) and Alzheimer's disease (14.8%). Hypertension was observed more frequently in men, but diabetes was observed more frequently in women (*p *< .05). An LP shunt was the first‐choice (55.1%) treatment of iNPH, followed by a VP shunt (43.2%).

**Conclusion:**

This study showed that iNPH occurs most frequently in the 70s, gait impairment and cognitive decline are the most frequent initial symptoms in men and women, respectively, and hypertension and diabetes are the most frequent comorbidities in men and women, respectively.

## 1 Introduction

Idiopathic normal pressure hydrocephalus (iNPH) is a treatable cognitive disorder with gait impairment, cognitive decline, and urinary incontinence as its three major symptoms. In iNPH, cerebrospinal fluid (CSF) pressure is normal despite ventricular dilatation, and symptoms are alleviated by a CSF shunt operation (Hakim & Adams, 1965). Since each of the above symptoms has negative effects on activities of daily living (ADL), its accurate and early detection, diagnosis, and treatment are important, and its clinical background and pathological features are attracting increasing interest (Bakker et al., 2002; Boon et al., 2000; Gallia, Rigamonti, & Williams, 2006).

There has been one previous report of a nationwide survey (Lemcke et al., 2016) to determine the epidemiology and clinical background of the disease, information which may be beneficial to both patients and doctors. In addition, because painful examinations such as the CSF tap test are necessary for definitive diagnosis of this disease, most past reports were limited to hospital‐based studies in patients at a limited number of hospitals. Furthermore, only a few epidemiological studies have been conducted, using standardized guidelines for the diagnosis and treatment of iNPH, and epidemiological information, such as incidence, has not been clearly presented.

According to previous retrospective analyses based on overseas epidemiological surveys, the prevalence of iNPH in local populations varied from 2 to 20/100,000 people (Brean & Eide, 2008; Vanneste, Augustijn, Dirven, Tan, & Goedhart, 1992). All reports had the limitation that iNPH was not definitively diagnosed in all subjects by a CSF tap test or shunt operation; thus, many patients with iNPH were likely to have been overlooked. In 2013, a nationwide epidemiological survey of patients who met the diagnostic criteria for iNPH was carried out primarily by the Study Group on Idiopathic Normal Pressure Hydrocephalus of Japan. In this survey, various epidemiological features of patients were investigated, including sex, age distribution, and various clinical symptoms and complications associated with this disease, and descriptive epidemiological analyses were performed.

## 2 Materials and Methods

### 2.1 Patients and methods for this nationwide epidemiologic survey

This study consisted of two epidemiological surveys. First, the total number of patients with a diagnosis of iNPH who received medical care during the year 2012 was estimated. Then, in the second survey to clarify the clinical features of this disease, the attending physicians of all patients registered in the first survey were asked to answer a questionnaire regarding specific clinical information and to mail it back to us.

### 2.2 Diagnostic criteria

This study was performed, using diagnostic criteria based on the Guidelines for Management of Idiopathic Normal Pressure Hydrocephalus: Second Edition, 2012 (Mori et al., 2012). Analysis was performed by dividing the patients into three diagnostic levels, possible, probable, and definite, according to the Guidelines.

### 2.3 Departments and medical institutions surveyed

The departments, lists of medical institutions, and special stratified hospitals were selected for the first and second surveys by the method standardized by the Research Committee on Epidemiology of Intractable Diseases in Japan, following previous nationwide surveys of other diseases (Fukushima et al., 2010; Nakamura et al., 2000).

Similar to the above reports, survey targets from all iNPH‐associated departments in Japan were selected by stratified random sampling. The hospital lists were widely available in the form of an electronic database and included all Japanese hospitals. Stratification was conducted according to the number of hospital beds. Sampling fractions were as follows: general hospitals with 99 or fewer beds (5%); 100 to 199 beds (10%); 200 to 299 beds (20%); 300 to 399 beds (40%); 400 to 499 beds (80%); 500 or more beds (100%); and university hospitals (100%). As special stratified hospitals for iNPH, 75 departments that treated numerous patients with iNPH were specially selected to have a 100% sampling fraction.

The first survey began in February 2012. The selected survey target departments were asked by mail to report the total number and sex of patients seen for iNPH between January 1 and December 31, 2012. Since the questionnaire was sent in February 2013, it was theoretically possible to collect the data for the total number of patients who visited for iNPH between January 1 and December 31, 2012. The methodology of the surveys published in the previous reports was followed exactly (Fukushima et al., 2010; Nakamura et al., 2000). Reminder letters were mailed to the departments that did not respond to the first survey.

### 2.4 Analysis of recovered questionnaires

Supplement (A,B) shows the questionnaires used. The institutions/departments that answered the first survey (questionnaire shown in Supplement A) and reported patients with iNPH were subsequently asked to answer the second questionnaire regarding the patients' details (Supplement B). Departments of neurosurgery, neurology, psychiatry, and internal medicine were selected as survey targets and asked in the first survey regarding the presence or absence of patients who fulfilled the diagnostic criteria at each department during the 1‐year period in 2012, the number of iNPH patients seeking medical care, and the estimated number of patients who underwent shunt operations. According to the results, the annual numbers of patients seeking medical care and undergoing shunt operations, as well as their age and sex distributions, were estimated.

In this survey, all selected medical centers were asked to follow the Guidelines for Management of Idiopathic Normal Pressure Hydrocephalus: Second Edition, 2012 (Mori et al., 2012), which was published and widely available in our country. According to the guideline, the CSF removal test is performed by removal of a small volume of CSF (approximately 30–50 ml) via lumbar puncture (tap test). A decision on a positive or negative response to the CSF removal test is primarily based on the clinical symptoms. There are several measurements for the clinical symptoms, including assessment tools for NPH symptoms, and the global assessment of the activities of daily living. Gait can be assessed quantitatively, using the 3‐m timed up and go test, or the 10‐m straight walk test. The mini‐mental state examination, frontal assessment battery, and/or trail‐making tests were applied for the assessment of cognition. The selected departments and medical institutions surveyed were strongly encouraged to ensure that all assessments complied strictly with the guideline.

If a department responded that it had a patient(s) in the first survey, a second survey consisting of a structured questionnaire was sent to collect data regarding the clinical characteristics of each patient. To avoid a low response rate, the questionnaire about the clinical characteristics was limited to an A4‐sized page.

### 2.5 Statistical analysis

Accounting for the selection rate and response rate of the survey, the total number of patients seeking medical care was estimated according to the following formula: estimated total numbers of patients = reported number of patients / (selection rate × response rate). Additionally, the 95% confidence interval (CI) was calculated with the assumption of a multinomial hypergeometric distribution (Fukushima et al., 2010; Nakamura et al., 2000). For the analysis of treatment by shunting, good responders were defined as those who showed improved modified Rankin Scale (mRS) scores. The group of good responders was determined by the number who showed obvious positive improvements, using the mRS score one year after the shunt operation**.** Subjects' characteristics were compared, using χ^2^ tests for categorical variables. Data were analyzed, using SPSS Inc. software version 19.0 (Chicago, IL, USA).

### 2.6 Ethical review

Ethical reviews of this study were completed at Juntendo University and Kyoto Prefectural University of Medicine, and approval was obtained in January, 2012. The study protocol was approved by the Ethics Committee of Kyoto Prefectural University of Medicine (Approval No.: E‐461).

## 3 Results

In the first survey, 4,220 of a total of 14,089 hospitals (459 university hospitals, 13,582 general hospitals, and 48 special stratified hospitals) were extracted, and an epidemiological investigation was carried out by mail. Formal answers were obtained from 1,804 hospitals, with a recovery rate of 42.7%. In the first survey, 3,079 patients who met the diagnostic criteria of iNPH and 1,815 patients who underwent shunt operations for iNPH were reported. From the data obtained in the first survey (Table 1), the number of patients treated for iNPH in 1 year was estimated, using the equation described in Methods to be 12,900 (95% confidence interval (CI): 10,000–15,800). Using this value, the crude prevalence of the disease in the Japanese population in 2012 was estimated to be 10.2/100,000. When iNPH is defined as the development of symptoms in the 60s or older according to the iNPH guideline, the crude prevalence of iNPH among those 60 years and older in 2012 was estimated to be 31.4/100,000. The estimated number of patients who underwent shunt operations for the treatment of iNPH was 6,700 (95%CI: 4,800–8,600). Thus, approximately 6,200 patients, that is, the difference between the above figures, were considered to have not undergone surgery, suggesting that approximately half the patients were not treated with shunt operations.

**Table 1:** Estimated number of iNPH patients from the results of the first survey

	Estimated number of iNPH patients	95% confidence interval
Estimated number of patients that met the diagnostic criteria of iNPH	12,900	10,000–15,800
Estimated numbers of patients who underwent shunt operation	6,700	4,800–8,600

**Figure 1:**
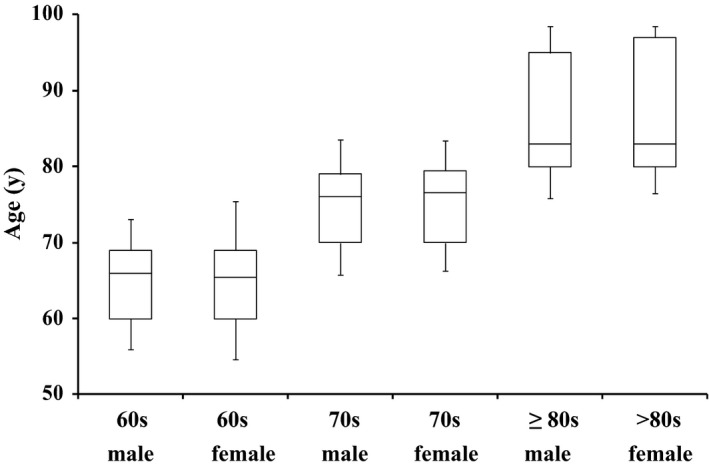
Age distribution of the subjects at the time of diagnosis. The age distribution of registered patients with iNPH at the time of definitive diagnosis is shown. Analysis is based on the subjects whose age at the time of diagnosis was available. Those in their 70s account for 50% or more of the registered patients, indicating the peak age of onset of this disease, followed by those in their 80s, accounting for approximately 30% of patients, and those in their 60s, accounting for 15% or less. The age distribution shows no sex‐associated characteristics or differences. These are descriptive statistics showing how the distribution by sex is similar. The box plots are based on the three‐number summary of age: minimum, median, and maximum. The segment inside the rectangle shows the median, and the bars above and below the box show the minimum and maximum, respectively

The estimated age of onset was in the 70s in more than 50% of the registered patients, showing a peak at this age range, in the 80s in approximately 30% of patients, and in the 60s in less than 15% (Figure 1). No significant difference was observed in the age of onset between the sexes.

Next, the clinical characteristics of the iNPH patients registered in the second survey were summarized. In the second survey, valid answers were obtained from 1,524 patients (Table 2). The mean age at the time of appearance of clinical symptoms and consultation with a medical facility was 74.9 ± 7.0 years, and that at which the patients underwent shunt operations for iNPH was 76.4 ± 7.0 years. The interval between the definitive diagnosis and shunt operation was approximately 1.5 years.

**Table 2:** Distribution of clinical background factors for all patients and by sex

Variable	All patients	Sex	
		Male	Female
	*n *= 1524 (100%)	*n *= 897 (58.5%)	*n *= 627 (40.7%)
	*n* (%)	*n* (%)	*n* (%)
Average age of iNPH patients			
Age at estimated onset (y)	74.9 ± 7.0	74.9 ± 6.7	74.8 ± 7.5
Age at diagnosis (y)	75.5 ± 8.6	76.4 ± 6.9	76.3 ± 7.3
Age at shunt operation (y)	76.4 ± 7.0	76.8 ± 8.9	76.9 ± 8.0
Current age at registration (y)	75.5 ± 8.6	75.3 ± 8.9	75.8 ± 7.9
Clinical Department of iNPH patients			
Neurosurgery	1179 (77.4)	685 (76.4)	494 (78.8)
Neurology	262 (17.2)	168 (18.7)	94 (15.0)
Psychiatry	60 (3.9)	32 (3.6)	28 (4.5)
General medicine	19 (1.2)	10 (1.1)	9 (1.4)
Others	4 (0.3)	2 (0.2)	2 (0.3)
Patients' current location			
1. Hospital	105 (6.9)	59 (6.6)	46 (7.3)
2. Ambulatory	816 (53.5)	487 (54.3)	329 (52.5)
Both 1 + 2	407 (26.7)	244 (27.2)	163 (26.0)
Deceased	19 (1.2)	15 (1.7)	4 (0.6)
Other	177 (11.6)	92 (10.3)	85 (13.6)
Diagnostic classification			
Possible iNPH	394 (25.8)	223 (24.9)	171 (27.3)
Probable iNPH	267 (17.5)	165 (18.4)	102 (16.3)
Definite iNPH	799 (52.4)	475 (53.0)	324 (51.7)
Unknown	64 (4.2)	34 (3.8)	30 (4.8)
Shunt treatment			
Shunt operation (+)	1004 (65.9)	594 (66.2)	410 (65.4)
VP shunt (% of operations)	434 (43.2)	248 (41.8)	186 (45.4)
LP shunt (% of operations)	553 (55.1)	334 (56.2)	219 (53.4)
VA shunt (% of operations)	17 (1.7)	12 (2.0)	5 (1.2)
PPV (% of operations)	990 (98.6)	587 (98.8)	403 (98.3)
DPV (% of operations)	9 (0.9)	5 (0.8)	4 (1.0)
Valve unknown	5 (0.5)	2 (0.3)	3 (0.7)
No shunting	464 (30.4)	271 (30.2)	193 (30.8)
Operation unknown, not filled in	56 (3.7)	32 (3.6)	24 (3.8)
Cause of death	29 (1.9)		
Pneumonia	6	5	1
Aspiration pneumonia	3	3	0
Cancer	6	5	1
Brain hemorrhage	3	2	1
Cerebral subdural hematoma	2	1	1
Other	9	4	5
Initial symptoms at 1st visit (multiple answers allowed)			
1. Gait disturbance	755 (49.5)	474 (52.8)^a^	281 (44.8)
2. Cognitive impairment	240 (15.7)	127 (14.2)	113 (18.0)^a^
3. Urinary incontinence	22 (1.4)	9 (1.0)	13 (2.1)
1 + 2+3.	185 (12.1)	112 (12.5)	73 (11.6)
1 + 2.	111 (7.3)	59 (6.6)	52 (8.3)
1 + 3.	50 (3.3)	28 (3.1)	22 (3.5)
2 + 3.	11 (0.7)	4 (0.4)	7 (1.1)
Other, unknown	150 (9.8)	84 (9.4)	66 (10.5)
Comorbidity			
Hypertension	609 (40.0)	383 (42.7)^a^	226 (36.0)
Diabetes mellitus	272 (17.8)	185 (12.1)	87 (13.9)^a^
Alzheimer disease	225 (14.8)	129 (14.4)	96 (15.3)
Hyperlipidemia	206 (13.5)	116 (12.9)	89 (14.2)
Lumbar spondylosis	154 (10.1)	85 (9.5)	69 (11.0)
Malignancy	82 (5.4)	54 (6.0)	28 (4.0)
Cervical spondylosis	49 (3.2)	31 (3.5)	18 (2.9)

VP shunt, ventriculoperitoneal shunt; LP shunt, lumbo‐peritoneal shunt; VA shunt, ventriculoatrial shunt; PPV, programmable pressure valve; DPV, (fixed) differential pressure valve.

a *p *< .05.

The diagnostic level was definite iNPH in 799 (52.4%) patients, possible iNPH in 394 (25.9%), and probable iNPH in 267 (17.5%). During the year, 29 patients with iNPH registered in this study died, and pneumonia, intracranial subdural hematoma, and cancer were the major causes of death.

Treatments recommended in the guidelines are VP shunt (ventriculoperitoneal shunt), VA shunt (ventriculoatrial shunt) and, if the patient shows no spinal canal stenosis and adequate CSF passage, LP shunt (lumboperitoneal shunt) (Marmarou, Bergsneider, Klinge, Relkin, & Black, 2005; Marmarou, Black, Bergsneider, Klinge, & Relkin, 2005; Marmarou, Black, Bergsneider, Klinge, & Relkin, 2005; Mori et al., 2012; Relkin, Marmarou, Klinge, Bergsneider, & Black, 2005; Sasaki et al., 2008; Toma, Holl, Kitchen, & Watkins, 2011). In this survey, LP shunt was the first choice (55.1%) of treatment for iNPH, followed by VP shunt (43.2%), suggesting that, at present, these two shunt operations are the mainstays for the treatment of iNPH.

The initial symptom observed at the first hospital visit was gait impairment alone in 49.5% of patients and cognitive decline alone in 15.7%; however, urinary incontinence alone was observed in 1.4%. Only 12.1% of the patients had all three classic symptoms, and the study showed that patients do not necessarily complain of symptoms that readily lead to a diagnosis of iNPH at the first examination. The initial symptom was significantly more often gait impairment in men and cognitive decline in women (*p *< .05).

Hypertension was the most frequent comorbidity with iNPH, seen in 40.0% of patients (Table 2). Diabetes was present in 17.8% of patients, Alzheimer's disease (AD) was present in 14.8%, and hyperlipidemia was present in 13.5%. Hypertension was observed significantly more often in men, and diabetes was observed significantly more often in women (*p *< .05). The characteristics of each stage of iNPH are shown in Table 3.

**Table 3:** Clinical background of iNPH patients by diagnostic level

Number	All patients	Possible iNPH	Probable iNPH	Definite iNPH	Unknown
	*n *= 1524 (100%)	*n *= 394 (25.9%)	*n *= 267 (17.5%)	*n *= 799 (52.4%)	*n *= 64 (4.2%)
	*n* (%)	*n* (%)	*n* (%)	*n* (%)	*n* (%)
Sex					
Male	897 (58.9)	223 (56.6)	165 (61.8)	475 (59.4)	34 (53.1)
Female	627 (41.1)	171 (43.4)	102 (38.2)	324 (40.6)	30 (46.9)
Clinical department of iNPH patients					
Neurosurgery	1178 (77.3)	257 (65.2)	181 (67.8)	694 (86.9)	46 (71.9)
Neurology	262 (17.2)	95 (24.1)	77 (28.8)	78 (9.8)	12 (18.8)
Psychiatry	61 (4.0)	34 (8.6)	9 (3.4)	13 (1.6)	5 (7.8)
General medicine	19 (1.2)	4 (1.0)	0 (0)	14 (1.8)	1 (1.6)
Others	4 (0.3)	4 (1.0)	0 (0)	0 (0)	0 (0)
Patients' current location					
1. Hospital	105 (6.9)	37 (9.4)	10 (3.7)	51 (6.4)	7 (10.9)
2. Ambulatory	816 (53.5)	221 (56.1)	158 (59.2)	414 (51.8)	23 (35.9)
1 + 2.	407 (26.7)	67 (17.0)	54 (20.2)	271 (33.9)	15 (23.4)
Deceased	19 (1.2)	8 (2.0)	3 (1.1)	8 (1.0)	0 (0)
Other (transfer, etc.)	177 (11.6)	61 (15.5)	42 (15.7)	55 (6.9)	19 (29.7)
Clinical symptoms at diagnosis (multiple answers allowed)					
Gait disturbance	1082 (71.0)	249 (63.2)	196 (73.4)	621 (77.7)	16 (25.0)
Cognitive impairment	532 (34.9)	154 (39.1)	94 (35.2)	281 (35.2)	3 (4.7)
Urinary incontinence	330 (21.6)	133 (33.8)	49 (18.4)	147 (18.4)	1 (1.6)
Unknown, not filled in	44 (2.9)	0 (0)	0 (0)	0 (0)	44 (68.8)
Comorbidity					
Hypertension	609 (40.0)	152 (38.6)	100 (37.5)	337 (42.2)	20 (31.3)
Diabetes mellitus	272 (17.8)	59 (15.0)	42 (15.7)	160 (20.0)	11 (17.2)
Alzheimer disease	225 (14.8)	91 (23.1)	47 (17.6)	78 (9.8)	9 (14.1)
Hyperlipidemia	206 (13.5)	47 (11.9)	31 (11.6)	117 (14.6)	11 (17.2)
Lumbar spondylosis	154 (10.1)	29 (7.4)	29 (0.4)	96 (12.0)	0 (0)
Malignancy	82 (5.4)	18 (4.6)	22 (8.2)	39 (4.9)	3 (4.7)
Cervical spondylosis	49 (3.2)	9 (2.3)	9 (3.4)	30 (3.8)	1 (1.6)

As for clinical symptoms at the time of definitive diagnosis, gait impairment, cognitive decline, and urinary incontinence were noted in 71.0%, 34.9%, and 21.6% of the patients, respectively, differing from the rates reported at the initial examination. However, no particular characteristics or differences were observed among the groups of iNPH patients. For all diagnostic levels of iNPH, hypertension was the most frequent comorbidity, and AD was not uncommon, observed in 23.1% of those with possible iNPH.

Table 4 shows the characteristics of examinations and treatments performed in patients at various diagnosis levels. Chronic cerebral ischemia noted on imaging studies of the brain may be a nonspecific finding associated with aging, but it is considered to have some importance.

**Table 4:** Clinical evaluation characteristics by diagnostic level

Numbers	All patients	Possible iNPH	Probable iNPH	Definite iNPH	Unknown
	*n *= 1524 (100%)	*n *= 394 (25.9%)	*n *= 267 (17.5%)	*n *= 799 (52.4%)	*n *= 64 (4.2%)
	*n* (%)	*n* (%)	*n* (%)	*n* (%)	*n* (%)
MRI findings					
Evans index ≥0.3	1259 (82.6)	321 (81.5)	216 (80.9)	701 (87.7)	21 (32.8)
Periventricular hyperintensity	882 (57.9)	247 (62.7)	168 (62.9)	457 (57.2)	10 (15.6)
Chronic ischemic lesion (diameter >1.5 cm)	142 (9.3)	43 (10.9)	25 (9.4)	73 (9.1)	1 (1.6)
CSF findings					
Elevation of CSF cell counts	17 (1.1)	2 (<0.1)	4 (1.5)	11 (1.4)	0 (0)
Elevation of CSF protein	203 (13.3)	43 (10.9)	50 (18.7)	106 (13.3)	4 (6.3)
Improvement of symptoms after CSF removal					
CSF tap test performed (+)	1273 (83.5)	251 (63.7)	249 (93.3)	719 (90.0)	54 (84.4)
CSF tap test: response (+)	1061 (83.3)	125 (49.8)	219 (88.0)	679 (94.4)	38 (70.4)
CSF tap test: response (−)	212 (16.7)	126 (50.2)	30 (12.0)	40 (5.6)	16 (29.6)
CSF tap test, not examined	179 (11.7)	114 (28.9)	10 (3.7)	47 (5.9)	8 (12.5)
CSF tap test, unknown	72 (4.7)	29 (7.4)	8 (3.0)	33 (4.1)	2 (3.1)
CSF drainage test (+)	48 (3.1)	9 (2.3)	7 (2.6)	28 (3.5)	0
CSF drainage test: response (+)	19 (39.6)	1 (11.1)	5 (71.4)	12 (42.9)	0
CSF drainage test: response (−)	29 (60.4)	8 (88.9)	2 (28.6)	16 (57.1)	0
CSF drainage test, not examined	1288 (84.5)	326 (82.7)	232 (86.9)	687 (86.0)	17 (26.6)
CSF drainage test, unknown	188 (12.3)	59 (15.0)	28 (10.5)	84 (10.5)	47 (73.4)

The CSF tap test, which is necessary for the diagnosis of probable or definite iNPH, was performed in 83.5% of the patients. Thus, the CSF tap test was confirmed as a standard CSF drainage test for the diagnosis of iNPH. Regarding CSF findings, it may be important for physicians to remember that a mild nonspecific increase in protein may occur in iNPH (Relkin et al., 2005). As shown in Table 5, approximately 55% of the patients were reported as improving after VP and LP shunting. The detailed subanalysis of good responders will be presented in a future report.

**Table 5:** Improvement by shunting operation

	Total numbers *n *= 1004	Numbers whose mRS scores improved (numbers with obvious positive improvement) (%)
VP shunting	434	238 (54.8)
LP shunting	553	309 (55.9)
VA shunting	17	12 (70.6)

## 4 Discussion

Idiopathic normal pressure hydrocephalus (iNPH) is an important geriatric disease and a treatable cognitive disorder, and its prevalence is expected to increase (Gallia et al., 2006; McGirt et al., 2008). While iNPH has been shown to be treatable by shunt operation, as its name suggests, its fundamental etiology or pathology has yet to be clarified. In 2012, the Guideline (Mori et al., 2012) proposed three diagnostic levels, possible, probable, and definite, and recommended treating the disease accordingly.

There has been only one previous descriptive nationwide epidemiological study (Lemcke et al., 2016) that reported the features and numbers of iNPH patients. In 2016, Lemcke et al. (2016) reported iNPH epidemiology based on nationwide insurance claim data from 2012. Although they reported that the prevalence of patients with iNPH in Germany was increasing rapidly between 8,609 and 11,363 for the last 5 years of the sample period (2008–2012), they did not report the precise nationwide prevalence in their paper. They mentioned that the annual incidence of definite iNPH treated by shunt surgery was estimated at 6.94/100,000 in 2012. However, the paper by Lemcke et al. was based only on insurance company claim data in Germany, unlike this study, which was based on clinical, hospital‐based data from Asia, which is useful for international comparisons. However, there have been no previous descriptive nationwide epidemiological studies that have provided accurate hospital‐based survey data on the clinicoepidemiological features of the disease, such as the total number of patients treated for iNPH and its current state of treatment. Therefore, accurate epidemiological information and nationwide epidemiological investigations are needed.

Outside of Japan, Vanneste et al. (Vanneste et al., 1992) reported the incidence of iNPH in the Netherlands as 2.2/1,000,000 people per year, and Brean et al. (Brean & Eide, 2008) reported the prevalence of suspected iNPH in Norway as 21.9/100,000 people. This result is similar to the present data for the crude prevalence of the disease. In Japan, the estimated prevalence of iNPH calculated only from the results of some community‐based studies has been previously reported. The prevalence of MRI‐supported possible iNPH was reported by Hiraoka et al. as 2.9% (Hiraoka, Meguro, & Mori, 2008). Iseki et al. (2009) reported that the incidence of iNPH above 60 years of age was 1.2/1000 persons per year. However, since all these reports were based on limited numbers of data obtained in restricted areas or special age groups, the data varied and did not provide conclusive epidemiological information allowing estimation of the number of patients from a national perspective.

Concerning the total number of iNPH patients and their classification as used in the present nationwide study, the number of patients who consulted medical institutions due to iNPH during the year 2012 was estimated to be 12,900 (Table 1). From this figure, the crude prevalence of the disease in 2012 was estimated to be 10.2/100,000 persons. Furthermore, 6,700 patients with definite iNPH were estimated to have undergone shunt operations in 2012. The present response rate of 42.7% was not as high as one would like, but this number is similar to previous reports and may thus be considered the minimum necessary. By adopting this uniform methodological method, the present data on the prevalence can be compared with other reports of iNPH or related diseases in the future.

However, it is important to note that the figures shown in this study, which was a nationwide hospital‐based survey, were no more than estimates due to the exclusion of patients who did not visit hospitals, and the true number of patients is likely greater. Therefore, the number of patients with iNPH was believed to not be small. This time, the criteria of iNPH including age of at least 60 years were followed. The main purpose was to understand the total prevalence of iNPH. Since the survey was performed in two parts, the respondents were not asked to provide information about all patients' ages in the first survey in order to have a simple and easy to answer survey. This was a difficult decision that was taken to avoid having a low initial response rate. Thus, exact information about age was not available from the first survey, which is a limitation when attempting to estimate the numbers of patients that met the diagnostic criteria of iNPH. However, if one can assume that the age distribution in the first survey was quite similar to that in the second survey, and then one applies the age distribution from the second survey to calculate the numbers with iNPH in the first survey, one can infer that the number of patients (70–79 years) treated for iNPH in 1 year was estimated to be 7,000 (95% confidence interval (CI): 5,400–8,600), and the number of patients (over 80 years) was also estimated to be 4,500 (95% confidence interval (CI): 3,500–5,600). Based on the above numbers of patients, the crude prevalence of iNPH among those 70–79 years and those over 80 years in 2012 could be 51.3/100,000 and 50.7/100,000, respectively. These results strongly suggest that the prevalence of iNPH increases with age, because it is a representative geriatric disease.

In addition, this study clarified that the symptoms of iNPH often appear in the mid‐70s, that patients begin to visit hospitals around this age, and that once the patients have consulted medical facilities, they are diagnosed and treated properly within 1.5 years. Interdepartmental cooperation for comorbidities and dispersion of information regarding appropriate diagnostic criteria and treatment guidelines is believed necessary in the future.

Among the diagnostic levels, definite iNPH was the most frequent, accounting for 52.4% of patients. Since the Guidelines recommend shunt operation for definite iNPH, more than half the patients had received aggressive surgical treatment. To promote awareness of the appropriate selection of shunt operations and to improve the clinical skills of physicians at outpatient clinics, further prospective investigations are warranted. Regarding the location of patient care, 53.5% were outpatients, and many patients hoped to return to normal activities after shunt surgery.

Regarding the clinical symptoms, according to the literature, gait impairment, cognitive decline, and urinary incontinence were observed in approximately 90–100%, 70–90%, and 50–80% of iNPH patients, respectively (Krauss et al., 1996; Mori, 2001). In this study, the frequency of patients with these symptoms was slightly lower, but gait impairment was found to be the most common, followed by cognitive decline and urinary incontinence. However, only 12.1% of the patients had all three symptoms; thus, iNPH should still be considered when examining elderly patients with a reduced ADL level, even if they lack some symptoms (Mori, 2001).

Next, the CSF tap test as a diagnostic method (Ishikawa, Hashimoto, Mori, Kuwana, & Kazui, 2012) was evaluated. However, some patients may be diagnosed with possible iNPH based on brain MRI findings and clinical symptoms, but not diagnosed with definite iNPH due to avoidance of the tap test or drainage, which is considered invasive and may carry risks for older patients (Marmarou, Bergsneider, Klinge, et al., 2005).

Recently, there has been increasing interest in the comorbidities of iNPH and their impacts on the evaluation of long‐term outcomes of iNPH patients (Malm et al., 2013). In the present survey, hypertension was the most frequent comorbidity (40% of patients), followed by diabetes mellitus (17.8%), AD (14.8%), and orthopedic disorders (approximately 13%). Previous reports noted that iNPH is often complicated by hypertension or secondary diabetes, clinically resembling vascular dementia (Andren, Wikkelso, Tisell, & Hellstrom, 2014; Graff‐Radford & Godersky, 1987; Jacobs, 1977; Jaraj et al., 2016; Krauss et al., 1996). Since lifestyle‐related diseases may be involved in iNPH as exacerbating or risk factors of gait and cognitive impairment, results of continued longitudinal investigations are awaited.

Next, the diagnostic characteristics of various examinations were evaluated. Regarding brain MRI studies, 82.6% of the patients had an Evans index ≥ 0.3, and ischemia around the lateral ventricles, which reflects cerebral arteriosclerosis, was an associated MRI finding in 57.9% of the patients, consistent with a previous report (Krauss et al., 1997) .

The primary pathologic change in iNPH is disturbed CSF circulation (Johanson et al., 2004), for which CSF shunt operations are primarily performed (Kazui, Miyajima, Mori, & Ishikawa, 2015). While a study demonstrated the effectiveness of LP shunting in 2015 (Kazui et al., 2015), the selection is presently made according to the patient's condition and surgeon's experience (Anderson, Grant, de la Paz, Frucht, & Goodman, 2002; Klinge, 2012; Klinge, Hellstrom, Tans, & Wikkelso, 2012; Klinge, Marmarou, Bergsneider, Relkin, & Black, 2005). As shown in Table 2, an LP shunt was the most frequent first‐choice treatment. While a VP shunt used to be the mainstay of treatment for iNPH, brain damage due to insertion of a tube into the brain is a risk. The results of this study suggest that an LP shunt, which does not involve puncturing brain parenchyma, is increasingly selected as the first choice to reduce surgical invasiveness. In this study, an LP shunt (55.1%) and a VP shunt (43.2%) were selected at comparable frequencies, with approximately 55% showing improved mRS scores, reflecting their degree of disability or dependence in daily activities.

It is important to note that half of the patients were not treated with a shunt (Table 5). However, there were no apparent differences in the clinical risk factors, such as hypertension, clinical symptoms, and radiological examination findings. Thus, the reason for lack of shunt treatment was likely related to other characteristics that may increase the risk of surgery generally or fear of shunt operations by both patients and attending surgeons. Since no evidence was obtained to explain it, we are planning to obtain information about the background characteristics of the patients who were not treated with a shunt though they had been reported as having iNPH as the next project in the near future.

Finally, the several limitations of this study should be considered. The first one is that the patients included in the survey were prevalent cases, because the reported subjects were patients already seeking medical care during the one‐year period. This may lead to a discrepancy in the estimation of the frequency of iNPH. The second one was that it was difficult to follow the patients of the hospitals that did not provide a response, because it was not possible to gain access to data of the hospitals that were uncooperative. Since the survey was performed in two parts, the respondents were not asked to provide information about all patients' ages in the first survey in order to have a simple and easy to answer survey. This was a difficult decision that was taken to avoid having a low initial response rate. Thus, this means that exact information about age was not available from the first survey, which is a limitation when attempting to estimate the numbers of patients that met the diagnostic criteria of iNPH. Thus, only limited data about iNPH were obtained. If we have a chance to organize a second follow‐up survey, we will attempt to address this problem. The third one is that this survey did not include all cases under investigation, but rather a small sample due to the limited resources and funding available; iNPH is not a rare enough disease to perform an all‐case investigation. The fourth limitation is that comorbid AD is sometimes difficult to distinguish from iNPH. However, as shown in the guideline, AD does not have MRI characteristics such as ventricular dilatation and tight convexity, which are necessary for iNPH. Moreover, diffuse atrophy in AD patients is different from the typical features of MRI in iNPH, since iNPH is not defined as diffuse brain atrophy. Of course, it cannot be completely guaranteed that this survey did not include any pure AD cases, because it was a self‐reported questionnaire of each doctor's diagnosis, and the MRI findings were not double‐checked. Since some cases of AD may resemble iNPH cases in the initial stage, a few pure AD cases may have been included as iNPH cases in this survey. However, because the guideline indicates that pure AD patients can be distinguished from iNPH cases by checking the typical characteristic clinical symptoms, such as wide‐based gait, or brain MRI characteristics, all board‐certified medical doctors were asked to follow the guideline strictly to select iNPH patients rather than pure AD patients. Therefore, pure AD pathology was eliminated as much as possible from this survey. Thus, these remained the major limitations of this methodology as a large‐scale nationwide survey. Moreover, future subanalyses of this study are expected to clarify the characteristics of nonresponders to shunt operations, the actual state of application of the indications for shunt operations, comparison of the complications between the LP and VP shunt groups, and the necessity for measures to reduce the burden of care.

In conclusion, this first nationwide epidemiological study reported the clinical characteristics of iNPH, diagnostic methods presently used in clinical situations, and treatments selected. The data obtained in this study are expected to be useful for the elucidation of fundamental mechanisms related to the etiology of iNPH and earlier formulation of therapeutic strategies.

## Conflict of Interest

None declared.
