# Supplementary material for: Nationwide hospital‐based survey of idiopathic normal pressure hydrocephalus in Japan: Epidemiological and clinical characteristics
Source: Brain Behav. 2017 Jan 27;7(3):e00635. doi: 10.1002/brb3.635 (PMC5346522; doi:10.1002/brb3.635)
Supplement: Supplementary file 1 [file BRB3-7-e00635-s001.doc]

Supplement A. First iNPH survey

Date of reporting (Year, Month, Day) ____________________
Name of institution ____________________
Name of division ____________________
Name of reporting Dr. name ____________________
1. The number of patients that met the diagnostic criteria of iNPH ____________
2. Of the above cases, the number of cases that underwent shunt operation ____________

Supplement B. Second iNPH individual questionnaire

Name of institution:
Name of reporting doctor
Address of institution:
Telephone number:
Name of division: 1. Neurosurgery 2. Neurology 3. Psychiatry 4. Internal medicine 5. Others ( )

Please fill in the correct numbers or select the correct answer for each question.

Gender	1. Male 2. Female	Date of Birth	years , month, day, age ( )	
Address of patient	1. Name of State ( ) Name of City ( ) 2. unknown			
Classification of iNPH	1. Possible iNPH 2. Probable iNPH 3. Definite iNPH			
Date of occurrence	1. Year, 2. month, 3. day , age ( )			
Date of diagnosis	1. Year, 2. month, 3. day, age ( )			
Date of shunt operation	1. Year, 2. month, 3. day, age ( ) 4 - Not examined			
Current status and treatment	1. Administration 2. Outpatient 3. Both Date of last hospital visit: year, month, age ( )<br>4. Transfer (Name of transferred hospital)<br>5. Death → Year, month, day<br>Autopsy: 1 .Yes 2. No 3 .Unknown, Direct cause of death: ( )<br>6. Other ( ) 7.unknown			
Family history	1. No 2. Yes [relationship: a. father b. mother c. brother d. sister e. child f. other ( )			
Initial symptoms	1. Gait disturbance 2. Cognitive impairment 3. Urinary incontinence 4. Psychiatric disorders 5. Others ( )			
Complications	Multiple selections acceptable<br>1. Hypertension 2. Hyperlipidemia 3. Diabetes mellitus 4. Cervical spondylosis 5. Lumbar spondylosis<br>6. Alzheimer disease 7. Cancer ( ) 8. Others ( )			
Clinical symptoms (within the observed period)	1. Gait disturbance	1. no 2. yes(mild) 3. yes(severe) 4. unknown		
	1-1. Decrease of gait width	1. no 2. yes(mild) 3. yes(severe) 4. unknown		
	1-2. Decrease of foot elevation	1. no 2. yes(mild) 3. yes(severe) 4. unknown		
	1-3. Wide-based gait	1. no 2. yes(mild) 3. yes(severe) 4. unknown		
	2. Cognitive impairment	1. no 2. yes(mild) 3. yes(severe) 4. unknown		
	3. Urinary incontinence	1. no 2. yes(mild) 3. yes(severe) 4. unknown		
	4. Psychiatric disorder	1. no 2. yes(mild) 3. yes(severe) 4. unknown		
	5. Other motor impairment	1. no 2. yes( ) 3.unknown		
	6. Hyper muscle tonus	1. no 2. yes(mild) 3. yes(severe) 4. unknown		
Radiological examination findings	1. Abnormal head CT findings	1. no 2. yes 3. not examined 4. unknown		
	2. Abnormal head	a. No<br>b. Yes →<br>c. Not examined<br>d. Unknown	Ventriculae dilatation	1. No<br>2. Yes<br>3.Unk<br>Evans index 1. >0.3 2. <0.3<br>PVH 1. No 2. Yes<br>Ischemic lesions 1. No 2. Yes<br>White matter lesions 1. No 2. Yes
	3. Abnormal	a. No<br>b. Yes →<br>c. Not examined<br>d. Unknown	1.Cervical lesion →<br>2.Limbar lesion →	1. No 2. Yes<br>1. No 2. Yes
	4. Scintigram of	a. No<br>b. Yes →<br>c. Not examined<br>d. Unknown	1.Decrease in frontal lobe lesion→<br>2.Decrease in corpus callosum lesion →<br>3.Decrease in Sylvain fistula lesion→	1. No 2. Yes<br>1. No 2. Yes<br>1. No 2. Yes
Laboratory examination findings	1. Abnormal CSF pressure	1. No 2. Yes ($/cmH_2O$) 3. Unknown		
	2. Elevation of CSF cell counts	1. No 2. Yes (/mm3) 3. Not examined 4. Unknown		
	3. Elevation of CSF protein	1. No 2. Yes (mg/dl) 3. Not examined 4. Unknown		
	4. CSF tap test (CSF removal test)	1. Not effective 2. Effective 3 .Not examined 4. Unknown		
	5. CSF drainage (continuous lumbar CSF drainage)	1. Not effective 2. Effective 3 .Not examined 4. Unknown		
**Treatment**				
Shunt operation	1. No 2. Yes → effect of shunting (1. no 2. yes 3. unknown) 3. Unknown			
Category of shunt operation	Ventriculo-peritoneal shunt (VP shu	1. No 2. Yes → effect of shunting (1. no 2. yes) 3. Unknown		
	Lumbar-peritoneal shunt (LP shunt)	1. No 2. Yes → effect of shunting (1. no 2. yes) 3. Unknown		
	Ventriculo-atrial shunt (VA shunt)	1. No 2. Yes → effect of shunting (1. no 2. yes) 3. Unknown		
Shunting system	1. PPV 2. DPV 3. Other ( ) 4. Unknown			
Complication	1. No 2. Yes ( ) 3. Unknown			

Abbreviations
PVH: Periventricular hyperintensity
CSF: Cerebral spinal fluid
PPV: Programmable pressure valves
DPV: Fixed differential pressure valve
